# Protein demand: review of plant and animal proteins used in alternative protein product development and production

**DOI:** 10.1093/af/vfaa040

**Published:** 2020-10-30

**Authors:** B Pam Ismail, Lasika Senaratne-Lenagala, Alicia Stube, Ann Brackenridge

**Affiliations:** 1 Department of Food Science and Nutrition, University of Minnesota, St. Paul, MN; 2 Protein Research & Development and Innovation, Cargill, Inc., Wichita, KS; 3 Starches, Sweeteners, and Texturizers Research & Development, Cargill, Inc., Minneapolis, MN

**Keywords:** alternative protein products, animal-based proteins, ingredients, plant-based proteins

**Implications**

- Increased global usage of protein has resulted in demand for protein products to surge over the last few years.
- Demonstration of equivalent or superior/new functions of novel proteins compared to existing alternatives is essential to protein market success.
- Additional investigation of nonprotein ingredients and innovation in production technologies for alternative protein products is necessary to continue the expansion of protein offerings in the market.

## Introduction

The demand for protein ingredients has surged over the last few years. The global protein ingredient market was valued at USD 38 billion in 2019 and is expected to grow at a rate of 9.1% from 2020 to 2027 (Grandview Research, 2020). Consumption of animal proteins has considerably increased in the recent past, as well as with a growing interest in overall protein, the market for plant protein ingredients is expected to grow significantly. Plant proteins can offset market share from animal proteins (dairy, egg, and meat) because they can be produced at competitive prices.

There are multiple factors driving the demand for proteins. The animal protein market will continue to grow because of the associated health benefits of consuming meat. Dairy and other animal proteins also play a major role in demand through diet supplements and food usage. Increases in the vegan, vegetarian, and flexitarian populations have propelled the usage of plant proteins in food products. Additionally, plant proteins are being used in manufacturing a wide range of natural products. Overall, the growing food industry on account of increasing population and consumer awareness is propelling the protein market and the need for alternative protein ingredients.

Furthermore, there is a global challenge to address food security and preserve land and water resources due to climate change, population growth, and changing diets. Accordingly, interest in sustainable and biodiverse food systems is on the rise. From a consumer’s perspective, purchasing habits that can improve the environment are gaining prominence. Consumers are seeking transparency and sustainability in their food supply. Accordingly, food industries are interested in commercializing products formulated with ingredients derived from environmentally sustainable crops.

Another important reason for seeking novel plant protein ingredients is protein allergenicity. Eggs, dairy, and soy are among the “big eight” major allergens recognized by the Food and Drug Administration. Other opportunistic reasons include utilizing current processing streams to increase the value and revenue (adding value to byproducts), finding a unique and competitive place in the market, and utilizing all possible resources to expand the ingredients supply. Additionally, producers are seeking functional, nonallergenic ingredients that can replace synthetic ingredients (such as synthetic emulsifiers, e.g., monoglycerides and diglycerides) as part of the clean label drive. Given that proteins have multiple functions, including, but not limited to, stabilizing properties, structure building, and flavor enhancement, producers are seeking to replace synthetic ingredients with functional proteins in various applications, including high-value ones, such as encapsulation of bioactive compounds and flavors (e.g., fish oil and orange oil).

Therefore, the demonstration of equivalent or superior/new functions of novel plant proteins compared to existing alternatives is essential to their market success. There is limited consumer and producer knowledge of plant proteins other than soy; nevertheless, new plant proteins are gaining traction, including pulse proteins (from pea, lentils, chickpeas, and beans) and proteins from canola, sunflower, oats, potato, rice, corn, and ancient grains among others (Grandview Research, 2016). Food producers are seeking to understand how these plant proteins can partially or wholly replace traditional plant and animal protein ingredients in food or plant-based meat-alternative products to deliver optimal nutrition, flavor, and functionality. Furthermore, advancement in nonprotein ingredient options and functionality are also in demand as these ingredients are combined with plant and meat proteins to fulfill the recipe needs (e.g., color, palatability, and shelf life) in the development of these food products.

While there has been some research done to characterize novel plant proteins, the information is far from being comprehensive. Science and technology must catch up with the exponential increase in the demand for novel plant protein. There is a need to explore efficient protein extraction processes to ensure high yields and preservation of the protein quality and functionality, understand structure/function relationship, develop cost-effective protein functionalization strategies, demonstrate ways to overcome flavor and texture challenges, identify unique high-value applications, investigate crop diversity, and secure abundant supply, along with evolving the nonprotein complimentary ingredients used in combination with the plant and animal proteins to satisfy the market demand. Our goal is to provide an overview of protein fundamentals and identify innovation needs and challenges across the protein supply chain to support demand surge in protein products.

## Proteins

**Figure 1.**
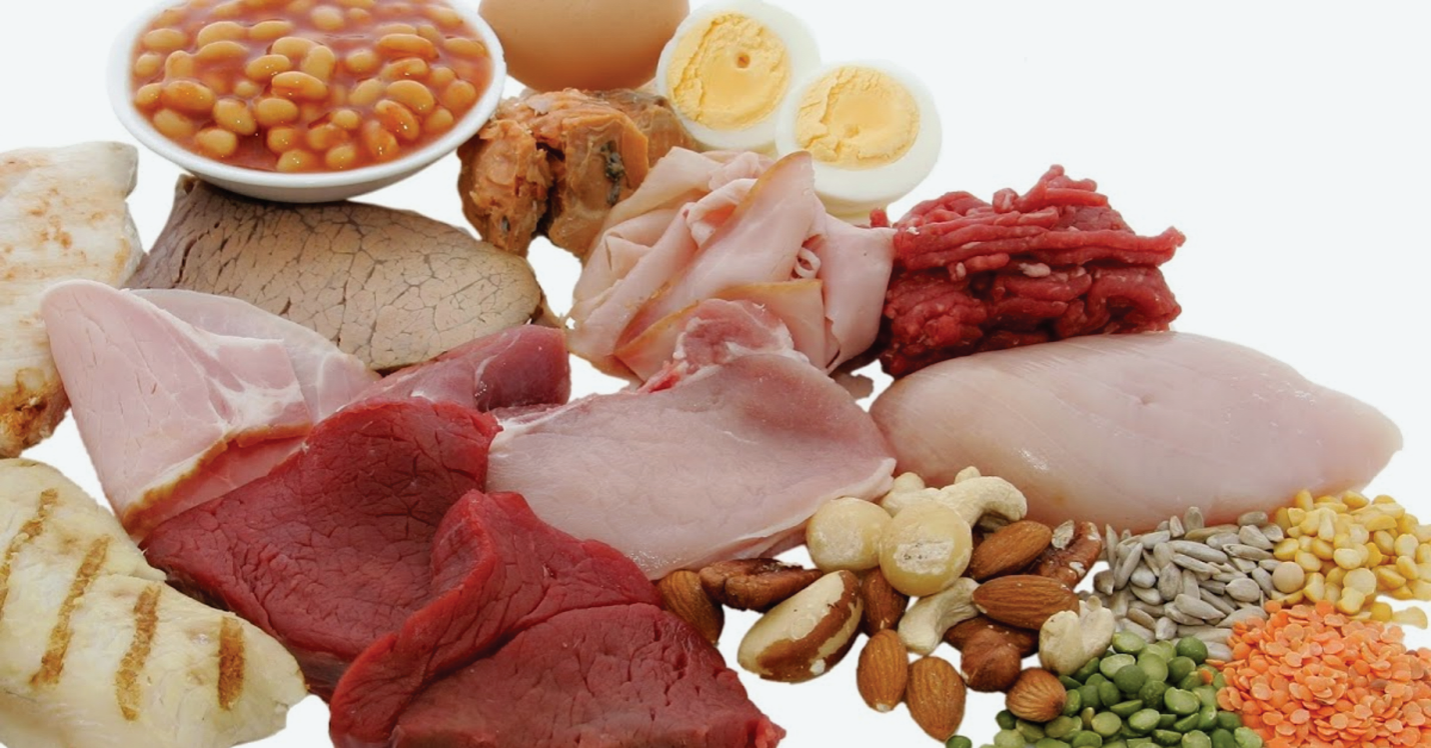
Animal- and plant-based protein.

Protein is a major and versatile constituent of food products (Figure 1). Apart from the nutritive value, the physicochemical and behavioral properties of proteins during processing play a significant role in determining the end quality of food. Due to the structural versatility and amphiphilic nature of proteins, they can interact with other food constituents, such as carbohydrates, fats, water, vitamins, minerals, and other proteins, through a range of interactions and bonds. In food production, animal and plant protein sources offer an array of functionality.

Common animal proteins used for processing in the food industry include the following: major milk proteins of casein and whey used for viscosity and stabilization of various food matrices; egg white protein used in forming networks for stability in whipping and heating of food products; and muscle proteins (myofibrillar, sarcoplasmic, and stromal) for applications ranging from gelation to color formation. Soy and pea are two plant proteins used broadly due to excellent functional properties, such as water holding, gelling, fat absorbing, and emulsifying capacities in food products. Gluten, a protein found in cereal grains, has unique cohesive and viscoelastic properties that can form fibrous proteinaceous networks and is commonly used in alternative meat products. Rapeseed and canola oil are oilseed proteins that are gaining attraction as ingredients for plant-based protein products. These proteins provide emulsification and foaming characteristics, as well as can form gels. Lentil, lupine, chickpea, pigeon pea, mung bean, and fava bean are other legume proteins studied on their physicochemical characteristics, including foam stabilization, emulsification, and gel formation. The overview of proteins provided is the surface of available plant and animal protein options and associated functionality for food producers.

## Protein Extraction Processes

**Figure 2.**
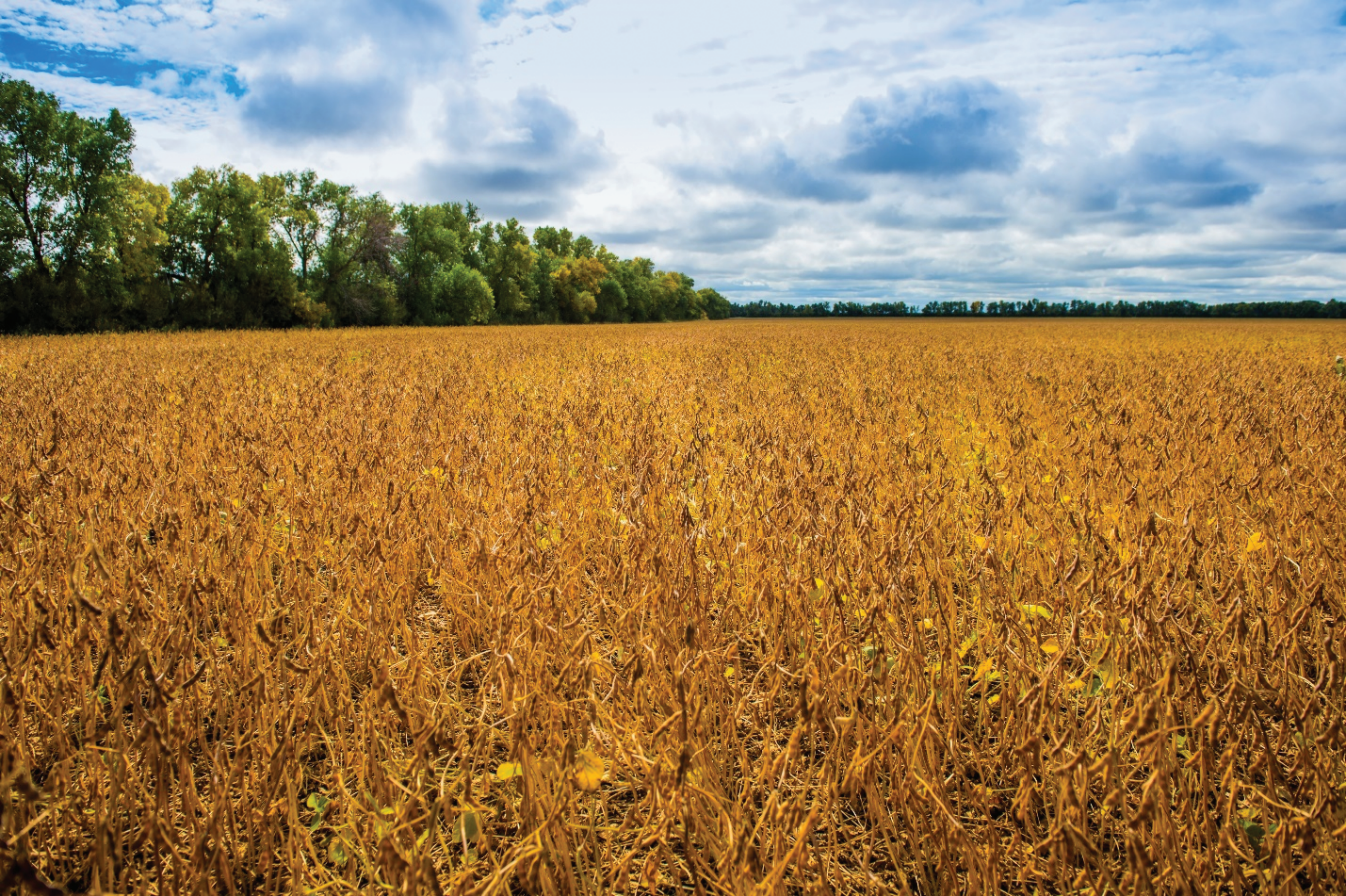
Soybean field in Manitoba, Canada.

Plant protein extraction and purification processes commonly begin with oil extraction, as is the case for oilseeds (e.g., soybean; Figure 2). Other initial steps in protein extraction are air classification to separate starch granules and fiber from protein bodies, as is the case for pulses, or steeping as in the corn milling process, which separates the corn into its four components, germ, fiber, starch, and protein. Cleaning and initial concentration steps for protein separation are crop dependent. Following initial separation and concentration, the protein-rich fraction is further processed to produce a protein concentrate (60–80% protein) or isolate (greater than 80% protein).

The protein from any given source is a heterogeneous mixture of different types of proteins. Therefore, purifying the protein following different methods will result in different protein profile, quality, and functionality. Protein purification can be done by the following methods of membrane filtration, chromatography, salt extraction, or pH solubilization/precipitation. For commercially available plant protein ingredients, namely soy and pea protein, the most common practice is pH solubilization/precipitation. The other purification processes, though may produce a protein ingredient that is more functional, such as chromatography, membrane filtration, or salt extraction, are more involved and costly.

Following pH-based extraction, the protein is solubilized at a pH (mostly alkaline, pH >7) where the protein is most soluble, while the starch and/or fiber will precipitate postcentrifugation. To separate the protein from soluble sugars and oligosaccharides, the protein is precipitated at its isoelectric point. The precipitate is washed, neutralized, and spray dried. Sometimes a diafiltration step is introduced prior to drying to reduce the amount of salt. The pH of solubilization may affect functionality, color, flavor, and digestibility. Low pH is often detrimental to the protein, causing denaturation and loss in functionality. Additionally, at alkaline pH, oxidation is favored, which can lead to browning and off-flavors in the presence of high level of polyphenols.

Therefore, it is important to optimize protein purification based on the source. Proteins from different sources have different structural characteristics that contribute to differences in their solubility and reactivity under various extraction conditions. Innovation in dry and wet extraction protocols are needed to enhance protein yield and purity while maintaining structural integrity and functionality.

## Structure/Function Relationship

The functional properties of the protein are dictated by the structural characteristics, including the amino acid composition and sequence, molecular size, and configuration, as well as physicochemical characteristics, such as surface hydrophobicity, net charge, and presence of reactive groups (e.g., sulfhydryl and hydroxyl groups). These characteristics can be interrelated; for example, the amino acid composition affects hydrophobicity and charge, while the sequence can affect molecular configuration, which, in turn, may affect surface properties. Surface properties affect protein solubility, thermal stability, and emulsifying and foaming properties, as well as gelation ability. For example, whey protein has very low surface hydrophobicity; therefore, it is highly soluble and is the golden standard for protein ready-to-drink beverages. On the other hand, proteins, such as soy protein, with high molecular weight and high surface hydrophobicity, may form polymers under specific conditions and can, thus, be texturized to form products with textural properties similar to meat products. Any change in the protein structure during purification and/or processing will impart a significant change in functionality.

## Functionalization Strategies

Often, protein powders are subjected to several functionalization processes, including agglomeration, lecithin coating, and high-pressure homogenization (Barbosa-Cánovas et al., 2005). These processes affect particle size, shape, and surface properties. Agglomeration increases particle size by forming bridges using binders, such as starch, gums, or hydrocolloids. This process enhances dispersibility, as water can diffuse easily within the agglomerate, while lecithin coating enhances wettability and prevents powder caking. High-pressure homogenization coupled with controlled spray drying conditions affects protein functionality. For example, high-pressure processing results in increased water-holding capacity and viscosity, desirable for meat-like applications. Powder functionalization through processing can be manipulated for targeted functionality enhancement. Different protein sources, however, may require unique processing approaches to enhance their functionality. A lot is known about soy and dairy protein functionalization. However, functionalization is an area that requires investigation for novel plant proteins.

Other functionalization strategies include protein-targeted modifications. The use of proteins in food formulations is subject to processing challenges due to their sensitivity to various processing parameters, including pH, temperature, shear stress, and enzymatic activity. Methods to improve protein functionality and stability during processing commonly focus on modifying the protein structure to improve solubility, increase flexibility, alter the hydrophilic/lipophilic balance, or promote protein cross-linking. The most commonly used protein modification in the industry is enzymatic hydrolysis.

Enzymatic hydrolysis is very well researched and is intended to improve functionality and provide physiological benefits. The degree of hydrolysis (%DH) and enzyme choice dictate the functional properties of the produced protein hydrolysate by influencing protein structure and peptide profile. A limited extent of hydrolysis (i.e., low %DH) is particularly important for producing functionally enhanced ingredients because it controls for both the loss in structure and release of bitter peptides associated with more extensive hydrolysis. Excessive hydrolysis (i.e., high %DH) results in a product high in free amino acids and short-chain peptides with minimal, if any, functionality. Limited enzymatic hydrolysis of soy protein (DH = 2–15%), for instance, resulted in increased solubility (Sun, 2011; Meinlschmidt et al., 2016), foaming (Tsumura et al., 2004), and emulsifying ability (Sun 2011; Meinlschmidt et al., 2016). Enzymatic hydrolysis needs to be optimized for each protein source to elicit the desired enhancement of a particular functionality.

Another protein modification approach is Maillard-induced glycation. Glycation is the addition of sugars to a protein or lipid. The effect of limited, controlled Maillard-induced glycation on improving protein functionality has been researched yet not commercially applied. The review by de Oliveira et al. (2016) highlighted 31 studies showing improved functionality for glycated proteins. Maillard-induced glycation may result in improved solubility, thermal stability, emulsification, foaming, and gelation properties due to increased hydrophilicity, viscosity, and protein cross-linking while lowering the protein’s isoelectric point and preventing denaturation (Wang and Ismail, 2012; Wang et al., 2013; de Oliveira et al., 2016;). However, the structural modifications and functional changes of glycated proteins depend on the Maillard-reaction conditions, protein conformation, and polysaccharide characteristics (e.g., chain length). Therefore, optimization of Maillard-induced glycation parameters is required to achieve the desired functionality of a particular protein while minimizing the propagation of the reaction to advanced and undesired stages (leading to browning and off-flavors). Furthermore, this technique needs to be made feasible for industrial application.

Nonthermal protein modification techniques, such as high pressure, oscillatory magnetic field, ultraviolet radiation, ozone treatment, pulsed electric fields, and, more recently, cold plasma, are gaining traction. Cold plasma technology involves the exposure of plasma, a partially ionized gas to proteins. The generated plasma may contain a range of reactive species, including electrons, positive and negative ions, and reactive oxygen, and nitrogen species, including free radicals at near room temperature conditions. The composition of reactive species is dependent on the gases used (e.g., air, O2, CO2, and Ar), reactor geometry, power application, and mode of interaction with the substrate to be treated (Ikawa et al., 2010). The different species can induce several chemical reactions, including oxidation, bond cleavage, and/or polymerization. Cold plasma is intensively used in industry for surface modification in material processing and ozone generation for water disinfection and is also investigated in the context of cancer treatment, wound healing, food decontamination, and blood coagulation (Inagaki, 2014; Mittal, 2014). The advantages of utilizing cold plasma include the preservation of quality attributes, cost-effectiveness, efficacy in reducing pathogens, short processing time, and lack of water and chemicals needed during processing. Cold plasma can be performed in open air and is adaptable, sustainable, and environment friendly (Ekezie et al., 2017). Few studies explored the cold plasma effect on the structure, functionality, and allergenicity of proteins from different sources (Tolouie et al., 2018). Studies did show changes in protein structure upon cold plasma treatment. However, findings were inconclusive due to the varied conditions tested, and results were not comprehensive in linking functional changes to structural modifications. Basic knowledge geared toward a better understanding of cold plasma modification is needed in order to develop a targeted approach to enhance plant protein functionality for desired applications.

## Crop Diversity and Supply

Currently, there is a gap between breeding crops to enhance yield and breeding to enhance functional and nutritional properties of the protein component. It is, therefore, crucial to investigate natural variations among existing lines not only in protein content but also in the protein profile and to develop markers and tools to initiate breeding strategies for direct enhancement in protein functionality and nutritional quality.

There are inherent differences in protein quantity and quality in different lines of a specific crop due to genetic variance, as well as environmental differences among the growing locations. A critical need for addressing the future utility of plant proteins in the food industry is identifying superior genetic variants for protein quality and functionality. This includes identifying accessions or varieties that currently have the best traits and identifying the genetic loci that can be used in breeding efforts to enhance these traits beyond their current usage. Specifically, identifying sources of germplasm with superior traits and the development of genetic markers will enable the efficient introgression of these traits into current and future breeding populations.

**Figure 3.**
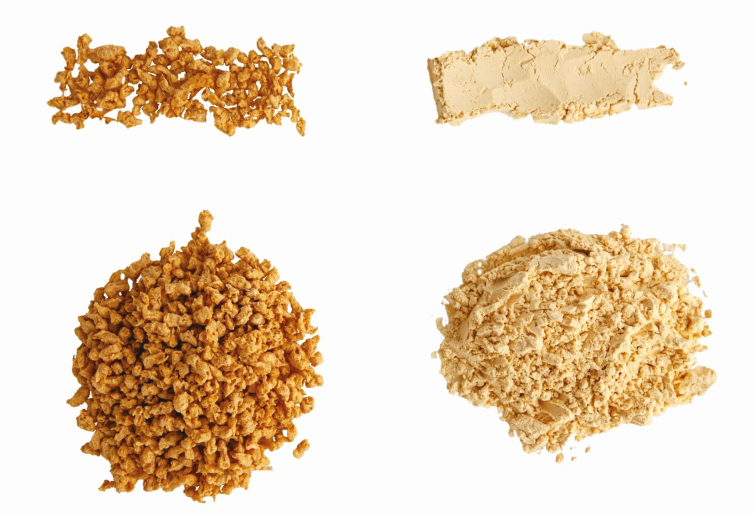
Textured pea protein and pea protein.

Other than breeding and genomics, research needs span agronomics, cropping system and agroecosystem design, efficient production of regenerative ecosystem services, and supply chain logistics. For example, short-season crops, such as pea (Figure 3), can be integrated in crop rotation to nourish the soil and provide additional revenue to farmers. For a novel plant protein source to be sustainable and abundant, a systematic approach needs to be employed to encompass the aforementioned research areas.

## Comparison of Animal and Plant Protein Quality

The nutritional quality of a protein is determined by its essential amino acid content, protein digestibility, net protein utilization, biological value, and protein digestibility-corrected amino acid score (PDCAAS; FAO/WHO, 1991). The PDCAAS is an indicator to assess protein quality by its ability to meet the human body’s amino acid requirements (FAO/WHO, 1991).

Animal proteins are more digestible, have greater net utilization, biological value, and PDCAAS than raw plant proteins (Table 1; Berrazaga et al., 2019). The low PDCAAS of plant protein sources could be due to lower digestibility and lack of certain essential amino acids needed for human body requirements.

**Table 1.** Protein quality assessment based on animal and plant protein sources; adopted from Berrazaga et al. (2019)

Protein type	% Protein digestibility	% Biological value	% Net protein utilization	% PDCAAS
Animal protein sources				
Beef	92	80	73	92
Chicken	95	79	80	91
Egg	98	100	94	100
Milk	96	91	82	100
Whey protein	100	104	92	100
Plant protein sources				
Soy flour	80	*N/A	*N/A	93
Soy protein isolate	98	74	61	100
Yellow split pea	88	*N/A	*N/A	64
Pea protein concentrate	99	65	*N/A	89
Chickpea	89	*N/A	*N/A	74
Wheat	91	56–68	53–65	51
Wheat gluten	85–95	64	67	25

*N/A—not available.

Animal proteins are more digestible compared to plant proteins (Table 1; Berrazaga et al., 2019). One reason is the structural differences between animal and plant proteins. Carbonaro et al. (2012) and Nguyen et al. (2015) found that plant proteins have more β-sheet structures and relatively low α-helixes than animal proteins, which makes them resistant to digestion in the digestive system. The presence of more fibers in plant protein is another reason plant-based protein has a lower proteolytic digestibility (Duodu et al., 2003). The presence of antinutritive factors is an additional factor for the lower digestibility of plant-based proteins in the human gastrointestinal tract compared to animal proteins. Most antinutritive factors are primarily found in protein bodies in cotyledon and the hull fraction of the legume seeds. Processing techniques can decrease antinutritive factor levels and increase the digestibility of plant protein (Tulbek et al., 2017). Understanding protein nutritive factors is essential in product development formulation strategies for alternative protein products to meet the human body’s protein needs.

## Plant Protein Flavor Challenges

The use of plant proteins, such as legume proteins, in food is challenging due to the persistent off-flavors that can be perceived by consumers. The off-flavors present in soy proteins are often described as “green,” “beany,” “painty,” and “grassy” (Rackis et al., 1979). These off-notes are commonly attributed to lipoxygenase-initiated peroxidation of unsaturated fatty acids (MacLeod and Ames, 1988) and are mostly attributed to the source of the raw material, processing, and/or storage. Pea flavor compounds have been investigated in raw, stored, and cooked peas (Malcolmson et al., 2014). Flavor compounds reported were saturated and unsaturated alcohols, aldehydes, ketones, alcohols, and their ester derivatives, as well as methoxypyrazines. Azarnia et al. (2011a) reported significant changes in volatile flavor compounds of peas during storage, while Azarnia et al. (2011b) reported differences in volatile compounds among cultivars and within cultivars grown in different crop years. To the best of our knowledge, there are no reports on flavor compounds retained in pea protein isolates or other novel plant protein ingredients. There is a need to design protein extraction/processing methods that yield neutral (bland) products. Masking off-aromas has been met with little success. Masking off-tastes, such as bitter, is possible but masking off-aromas is more complex due to how aroma is the sum of a pattern of the responses of numerous receptor types in contrast to taste, which typically deals with a single receptor. Accurate flavor profiling will lead to identifying approaches that eliminate the problematic off-flavors instead of attempting to mask them.

## Nonprotein Ingredients and Functions

### Texturizers

Texturizers used in food products act as water and oil binders, sliceability enhancers, filler or extenders, and texture and gelation enhancers in the finished product. Selecting animal- or plant-based texturizing ingredients is based on the product’s claim or targeted diet type. For example, an alternative meat product for flexitarians can incorporate both animal- and plant-origin binding and texturizing agents, such as soy protein isolates and concentrates, wheat gluten, milk proteins, egg whites, carrageenan, xanthan gum, methylcellulose, flours/starches, pectin, and other plant-based fibers and gums offering the greatest selection of functionality. Alternatively, in vegan or 100% plant-based claimed products, animal-based binders and texturizers, like milk proteins and egg whites, cannot be used. Vegan products typically rely on plant-based texturizers. However, egg white has been commonly used in food production as a binder due to its ability to form a firm, irreversible gel upon cooking. To meet the needs of the multiple diet types, further investigation into plant-based texturizers that offer greater functionality is necessary.

### Methylcellulose

Methylcellulose is a cellulose derivative produced by forming an alkali cellulose (reacting methyl chloride and alkali cellulose) that has distinctive gelation characteristics. It forms a thermo-reversible hard, brittle gel upon heating but reverts to a viscous liquid when cooled. In contrast, starches and hydrocolloids form thermo-reversible gels in the opposite direction—gelling when cold and melting back to a liquid when heated. This unique characteristic of methylcellulose makes it invaluable for providing binding and gel structure for foods served hot. The emulsification capacity of methylcellulose also helps prevent fat separation and increases the perception of succulence. In the development of plant-based food products, methylcellulose is prized for its versatility in functionality and role in product structure and eating experience. There is a continued need in the industry for vegan ingredients that can create a firm bite and juicy meat-like texture.

### Carrageenan

Carrageenan is a high molecular weight linear polysaccharide isolated from red seaweed. There are three basic carrageenan types: kappa produces a strong gel with potassium ions; iota forms elastic gels with calcium salts; and lambda forms thickened liquids and does not gel. When a heated solution of kappa carrageenan is cooled below its gelation temperature (30–70 °C depending on formulation conditions, such as the presence of salts), it will form a firm, brittle gel (Blakemore and Harpell, 2010) and is commonly used in meat products. This functionality improves slicing capability and texture in meat analog products, such as deli meats, served at or below room temperature. Carrageenan also has excellent water-binding capabilities and helps retain moisture for an improved eating experience.

### Starches

Starches in meat analogs act as fillers and enhance texture via their ability to bind and retain moisture. When heated in the presence of water, gelatinization occurs and the starch granules swell, entrapping the water released (process of breakdown of bonds) from the textured protein or other components of the formula (gelation, on the other hand, is the process of formation of a gel). Starches are available from a variety of botanical sources and in native and modified forms. Common starch modifications can improve freeze-thaw stability, decrease gelatinization temperature, or alter the viscosity (Joly and Anderstein, 2009). Critical consideration of the application is required to select one with the appropriate functionality. For example, a starch with a gelatinization temperature above the temperatures experienced during processing will not be able to contribute much functionality. Cold swelling starches may be used to build viscosity and bind water in an uncooked system. Overall, there are multiple starch options available. Starch selection for food product formulation is dependent on the needed functionality and how the product is prepared.

### Fiber ingredients

Fiber is a type of carbohydrate found in many foods, such as legumes, as well as whole grains and most vegetables and fruits. Fiber ingredients are used in plant-based products to add body and improve mouthfeel, as well as for their water-holding capacity. They also provide upfront viscosity and cohesiveness to help the product matrix hold up to handling and forming. Because of the breadth of fiber sources, the offering of fiber ingredients in the market for food production is broad.

### Fats

In traditional and alternative (cell- or plant-based) meat, as well as in plant-based products, fat contributes to the perceived tenderness and juiciness of the product and aids in flavor retention/release. Liquid oils contribute lubricity and add in the consumer perception of moisture, while saturated fats more closely mimic the fatty acid profile of traditional meat and contribute firmness to the chilled mix. Flaked solid fat can also add the expected appearance of marbling. Some plant-based fat options include vegetable oil, coconut oil, palm oil, and cocoa butter. The right combination of fats is important to achieve a desirable succulent mouthfeel and flavor linger.

### Flavorings

The flavor and taste of products are very important as they determine the overall consumer acceptability of the finished product. Savory, meaty, and metallic notes (iron or ferrous) are considered mainly in meat-alternative formulations to mimic real meat products. To attain savory and meaty flavors and aromas, some sulfur-containing amino acids (cysteine, cystine, and methionine), nucleotides, reducing sugars (like glucose, fructose), vitamins (thiamin), and other amino acids (proline, lysine, serine, methionine, and threonine) are commonly used as ingredients in alternative protein processing (Moon et al., 2011; Kyriakopoulou et al., 2019). Hydrolyzed vegetable proteins are another ingredient used in alternative protein product formulations to provide chicken- or beef-like aromas and flavors. Moreover, in alternative egg preparations, Himalayan black salt or “Kala namak,” which has a unique eggy-like taste and smell, due to its higher sulfur content, is commonly used to mimic real egg flavor and smell. Overall, flavor is integral to the consumption experience.

### Colorants

Color is a factor in the visual appeal of food. Colors in plant-based burgers, sausages, and minced meats are used to mimic red-pink color at raw state and brown when cooked. For those products, a combination of heat-unstable colorants and reducing sugars are used (Hamilton and Ewing, 2000). Thermally unstable pigments of betanin-pigment containing beetroot powder or juice are commonly used. Reducing sugars used in plant-based products are xylose, arabinose, galactose, mannose, dextrose, lactose, ribose, and maltose (Hamilton and Ewing, 2000) and can undergo a Maillard-type reaction with amino group of proteins during cooking, producing the brown color components. For items like plant-based hotdogs and hams, red-pink color in the final product is desirable. Heat-stable pigments or their combinations, such as annatto, turmeric, saffron, carotene, cumin, caramel color, paprika, red yeast rice powder, canthaxanthin, and astaxanthin, are often used to achieve the desired color since the red color does not degrade during heating. Most heat-stable and heat-labile colorants have an optimum pH range for higher-quality color; therefore, some level of pH adjustment with acidulent (acetic acid, citric acid, and/or lactic acid) is required in the final product formulations. The usage of acidulants is not always possible as they negatively affect the texture and flavor of the product (Kyriakopoulou et al., 2019). Recently, soy leghemoglobin, a plant-based heme-containing protein, is also used as a coloring agent in plant-based burgers to give “bleeding” appearance like in animal-based meat burgers. This pigment is denatured and converted into brown color upon cooking similar to myoglobin in meats.

The expansion of functionality and breadth of texturizers, fats, flavorings, and coloring agents are needed to evolve alternative protein product development. Hence, further investigation and development of the nonprotein ingredients are essential for consumer adoption and continued growth in the demand for food or plant-based meat-alternative products.

## Current and Future Technologies for Processing Alternative Protein Product

### Processing techniques

**Figure 4.**
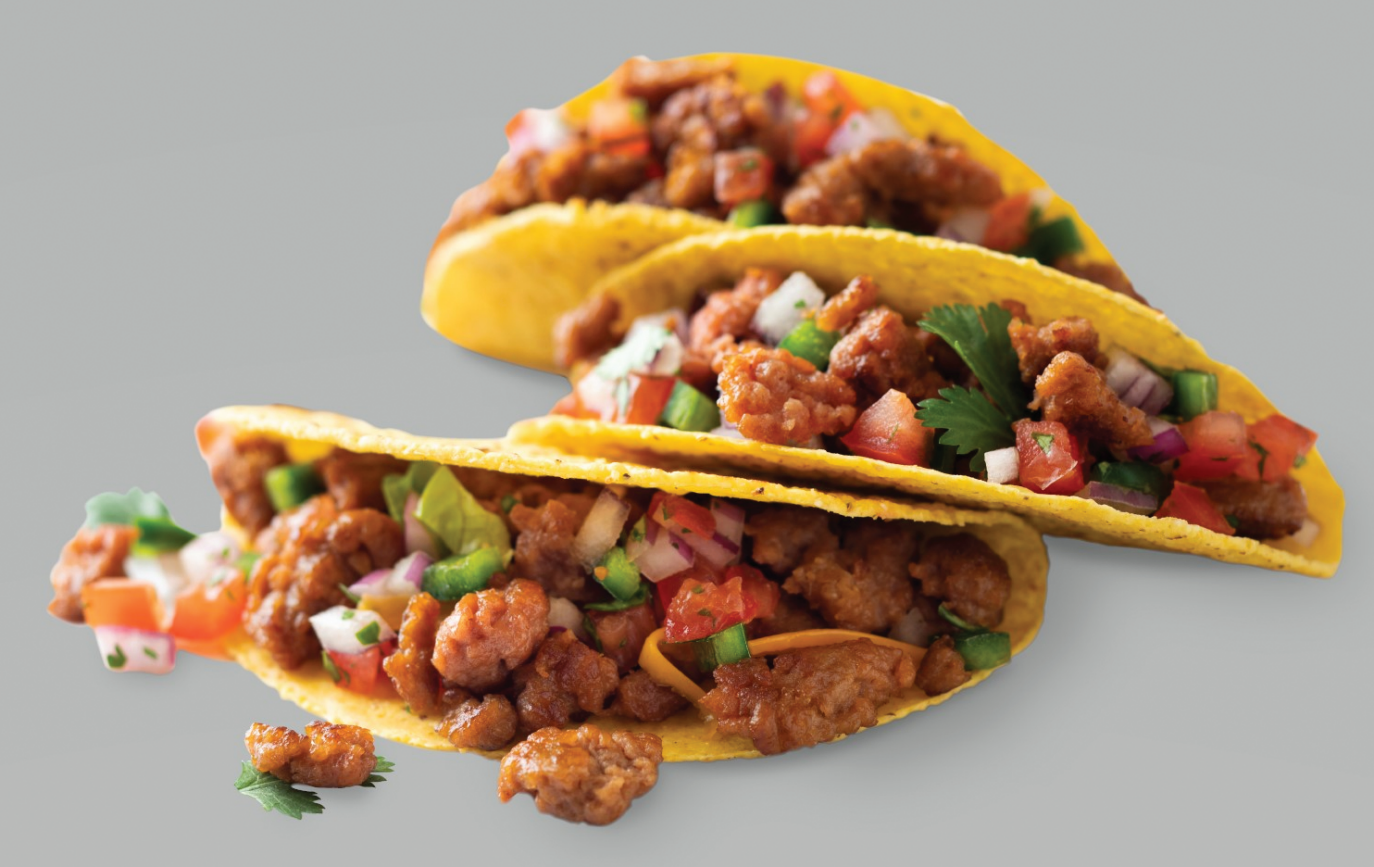
Plant-based grind.

One objective of alternative meat production is to have consumers perceive that they are eating meat products by mimicking the structure, composition, appearance, and flavor of animal protein products (Figure 4). The complex structure of the meat is challenging to reproduce with plant-based ingredients. Therefore, the search for plant proteins that provide nutritional and functional properties similar to animal proteins has continued at an increasing pace. Also, food technologists developing protein products have continuously focused on processing/structuring techniques with plant proteins that offer desirable sensory characteristics in 100% plant-based products, as well as provide appearances and eating sensations similar to meat counterparts.

Traditional plant-based alternative protein products are produced with simple processing techniques, such as fermentation, chemical-based protein coagulation, pressing, heating, steaming, cooling, and washing (Malav et al., 2015). Extrusion, shear cell technology, and 3D printing are developed modern processing techniques. There is a continued emphasis on improving these processes, as well as exploring other applicable protein-processing technologies.

### Extrusion

Extrusion is a common practice and extensively used to convert 50–70% protein-containing plant-based materials to fibrous products. It is a thermomechanical process that uses a combination of pressure, heat, and mechanical shear (Kyriakopoulou et al., 2019). There are several plant protein raw materials currently used as ingredients for extrusion, such as defatted soybean meal, soy protein concentrate and isolates, wheat gluten, pea protein concentrate and isolate, and peanut protein (Kyriakopoulou et al., 2019).

There are two types of extrusion processes based on the amount of water added during the process; low-moisture extrusion (20–40% moisture added) and high-moisture extrusion (40–80% moisture added). Low-moisture textured proteins must typically be rehydrated prior to use, often in combination with other ingredients. High-moisture extruded products may not require any further processing prior to use.

Important functional characteristics of extruded products are water and oil absorption (if in a low-moisture format), density, and size/shape. These characteristics are a factor of the initial feed material, extrusion conditions, die selection, and secondary cutting. A less-dense piece, such as a flake, will rehydrate more quickly than a mince but may sacrifice some firmness. Products with too much expansion will have a hard time retaining their structure after rehydration and can turn to mush during further processing or eating. Products with too little expansion will be very slow to rehydrate and may be perceived as a hard chunk with no distinguishable texture.

Preconditioning is an important initial step in protein extrusion to allow moisture to uniformly penetrate the protein particles prior to introduction into the extruder. In the extruder, proteins are subjected to high temperatures and pressures that cause the proteins to melt and denature (Zhang et al., 2019), losing their tertiary or even secondary structure. The denatured proteins realign in the direction of flow as they move through the screw, exposing bonding sites that allow the proteins to cross-link in a new way. This cross-linking is what texturizes the proteins and transforms globular plant proteins into structures that more closely resemble the fibrous and laminar construction of meat. As the material exits the die at the end of the extruder, the water in the mixture rapidly evaporates due to the high temperatures and release of pressure, causing the material to expand and creating the final puffed format. The design of the die has a significant impact on the shape and texture of the created pieces. Additionally, the material may be further cut to achieve the desired piece size and shape.

In addition to creating a meat-like structure, extrusion can also modify the color and flavor of protein components. Many undesirable flavors are volatile and will flash off along with moisture at the release of pressure at the end of the extruder. Extrusion may also improve the nutritional quality of the proteins. Extrusion process has been studied broadly for many decades now; however, the control over the process is one of the largest challenges (Zhang et al., 2019) and the design of extruded products is still not fully defined.

### Shear cell technology

The shear cell technology was introduced by a group of researchers at Wageningen University, Netherlands, around 2005 (Manski et al., 2007). It is another technique where a combination of shear and heat are used to form plant-based meat analogs, with layered fibrous structures, that resemble the mouthfeel and texture of real meat steak. The shearing device used in this technology is called shear cell, where intensive shear can be applied. There are two kinds of shear cells: conical cell based on cone-plate rheometer and cylindrical shape-Couette cell, which was developed for a scaling-up process (Manski et al., 2007). In this technology, the finished product structure depends on the ingredients and the processing parameters. Protein deformation in the shear cell is well defined and constant, mechanical energy input in structuring is low; therefore, the shear cell technology has less variation in product quality compared to extrusion (Manski et al., 2007; Krintiras et al., 2016). By increasing the size and length of the Couette cell, the device capacity and throughput can be increased. Several plant-based protein combinations (soy protein concentrate, soy protein isolate and wheat gluten, or soy protein isolate and pectin) were tested for their ability to form fibrous structures in shear cell technology (Manski et al., 2007; Dekkers et al., 2016). However, plant-based meat-alternative products made with shear cell technology are not commercially available.

### 3D printing

An innovative and versatile digital technology is 3D printing, which can be used for additive manufacturing and rapid prototyping. The 3D printing process can recreate a muscle-like matrix through micro-extruding filaments using a plant-based paste. The paste is placed in the 3D printer matrix with the help of Auto Computer-Aid Design (AutoCAD) modeling software (Carrington, 2020).

NOVAMEAT, one of the food technology companies making 3D printed plant-based meat products, has announced that they are able to recreate a steak with a firm, fibrous texture and meaty appearance using pea protein, rice protein, seaweed, rapeseed fat, and beetroot juice (Carrington, 2020). Redefine Meat is another company located in Israel that claims to produce alternative meat products, which mimic the appearance, texture, and flavor of animal muscle meat (Askew, 2020). The speed and variety of substrates used in 3D printing pose a significant opportunity for application in food development.

These evolving technologies expand the tools available to plant-based manufacturers to replicate and enhance the flavor, texture, and eating experience of products. They pave the way for greater versatility in the next generation of alternative protein food products and only represent the tip of the iceberg in a space ripe for innovation.

## Conclusions

The global demand for protein is projected to continue to grow. Protein quality and functionality differences remain between animal and plant proteins. The science and technology used across the supply chain of various protein products must catch up with the exponential increase in the demand for novel protein sources. To meet both consumer’s demand and desired eating experience, the expansion of options and functionality of nonprotein ingredients is essential for product development and manufacturing. Both plant and animal proteins are vital to meet the world protein supply needs.

*Conflict of interest statement.* None declared.

## About the Author

**Dr. B. Pam Ismail** is a Professor at the Department of Food Science and Nutrition, University of Minnesota. She is also the founder and director of the Plant Protein Innovation Center. Dr. Ismail has over 20 years of experience in food chemistry research focused on analytical chemistry, protein chemistry, enzymology, and chemistry and fate of bioactive food constituents. Her research focuses on chemical characterization and enhancement of functionality, safety, bioavailability, delivery, and bioactivity of food proteins and phytochemicals, following novel processing and analytical approaches. Dr. Ismail is the recipient of a “Distinguished Teaching Award” and an “Outstanding Professor Award.”

**Dr. Lasika Senaratne-Lenagala** is a Senior Food Scientist working at Cargill Inc., Wichita, KS in the Protein Research and Development (R&D) Group. She received her BS degree in agriculture from the University of Peradeniya, Sri Lanka, MS in biochemistry from Pukyong National University, South Korea, and her PhD in animal science (meat science and muscle biology) from the University of Nebraska-Lincoln. Her current R&D work focuses on both meat and plant/alternative protein product development.

**Alicia Stube** is a Senior Scientist in Cargill’s North American Starches, Sweeteners and Texturizers division, Minneapolis, MN. At Cargill, she focuses on research and development in the area of plant-based protein ingredients. Her current work includes understanding the functionality of established and emerging nonanimal proteins in food formulations. Alicia has 10 years of experience in the food industry in ingredient and finished product development and holds an MS degree in food science from Purdue University, West Lafayette, IN.

**Dr. Ann Brackenridge** is a Manager at Cargill Inc., Wichita, KS, in the Protein Research and Development (R&D) Group. She received her PhD from Kansas State University, Manhattan in muscle growth and development. Ann has over 20 years of R&D and food safety experience in the protein industry. Dr. Brackenridge has held prior positions in Cargill overseeing R&D teams working on product and technology development across meat animal species, as well as food safety plant operation’s teams in North America. Her research interests include improving meat quality and developing new protein technologies. Ann is currently President-elect, American Meat Science Association.
